# Medical Meetings

**Published:** 1895-05

**Authors:** J. McFadden Gaston


Medical meetings of special interest have been held in the city of Baltimore, during the past week. The American Academy of Medicine, the American Medical Association, the American Medical College Association, the American Medical Editors Association, and the American Medical Publishers Association, filled in the time from May 4th to May 10th. These were interspersed with festivities of a public and private nature, which added greatly to the interest and enjoyment of the visitors. While the scientific work occupied all the attention of the doctors during the day, the receptions and entertainments delighted both ladies and gentlemen during the evenings.

The following officers of the American Medical Association were elected:

President—R. Beverly Cole, of San Francisco.

Vice-Presidents—Julian J. Chisolm, of Baltimore; John C. LeGrand, of Alabama; Augustus B. Clark, of Massachusetts; T. J. Satterwhite, of Kentucky.

Treasurer—Henry P. Newman, of Chicago.

Secretary—W. B. Atkinson, of Philadelphia.

Assistant Secretary—J. McFadden Gaston, Jr., of Atlanta.

Librarian—G. E. Weiss, of Illinois.

Trustees—Alonzo Garcelon, of Maine; I. N. Love, of Missouri; James E. Reeves, of Tennessee.

Judicial Committee—N. S. Davis, of Chicago; H. D. Didama, of New York; John Morris, of Maryland; W. E. B. Davis, of Alabama; George W. Broome, of Missouri; D. W. Smouse, of Iowa; M. B. Ward, of Kansas; W. M. Welch, of Pennsylvania.

Dr. Atkinson begins his thirty-second term as permanent secretary. The nominating committee proposed to replace him by naming Dr. Frank Woodbury, of Pennsylvania. By unanimous vote, the Association refused to adopt the committee's recommendation, but re-elected Dr. Atkinson.

Another contest was over the selection of the next meeting place. Atlanta was named, but several Washington delegates said the Association had previously pledged itself to visit Washington in 1896. Dr. Jenner's discovery of vaccination is to be celebrated next May, in Washington, and several foreign physicians have been invited to attend the celebration, with the expectation that the American Medical Association would hold its meeting at the same time. The friends of Atlanta proved stronger in numbers, and that city was selected.

Next year, the annual addresses will be delivered as follows:

On "Surgery," by Dr. Nicholas Senn, of Chicago.

"Medicine," Dr. William Osler, of Baltimore.

"State Medicine," Dr. George H. Rohe, of Baltimore.

Dr. W. F. Westmoreland, of Atlanta, was appointed chairman of the committee of arrangements for the Atlanta meeting.

They had quite an exciting issue, in Baltimore, before the American Academy of Medicine, on the paper of Dr. Woods Hutchinson, upon the "Economics of Prostitution," on Saturday; and another paper, by Dr. Rochester, upon sexual physiology, on Monday, which were discussed together afterwards. An allusion to the former, by Dr. Walk, of Philadelphia, at the banquet, on Saturday night, with an unfavorable criticism, was resented in the reply of Dr. Hutchinson, on Monday, and he was sustained generally by the fellows of the Academy. Dr. Helen Putnam, vice-president of the Academy, joined in the discussion, and thus manifested the propriety of considering this class of questions. I made some remarks at the close of the discussion, endorsing the propriety of considering these delicate matters, but space does not permit any details.

In my address as president of the American Academy of Medicine, at Baltimore, an allusion was made to the plan of a four years graded course proposed by the American Medical College Association, and I am willing to go on record as follows:

It is a matter for serious consideration whether any considerable number of the medical colleges in the United States are in a condition to enter upon a four years graded course at the present time or within the near future. While the endowed institutions are prepared to assume the responsibilities of such a step, and it is commendable in independent organizations to afford the very best opportunities for the highest attainments of those who can devote this additional time to a preparatory training for the medical profession, a large majority of the medical colleges cannot undertake more than a three years graded course. They are ready to make a decided advance by the adoption of a three years graded course; and many of them have already entered upon the curriculum of instruction on this basis. This is so marked an improvement upon the old two years course that we should congratulate the profession upon this evidence of progress, and be content to await further developments to authorize an increase of another year. We should be satisfied to let well enough alone and not run the risk of disaster by such a great change so suddenly.

J. McFADDEN GASTON, M. D.

